# Investigation of the Efficacy of a Postbiotic Yeast Cell Wall-Based Blend on Newly-Weaned Pigs under a Dietary Challenge of Multiple Mycotoxins with Emphasis on Deoxynivalenol

**DOI:** 10.3390/toxins12080504

**Published:** 2020-08-06

**Authors:** Debora Muratori Holanda, Alexandros Yiannikouris, Sung Woo Kim

**Affiliations:** 1 Department of Animal Science, North Carolina State University, Raleigh, NC 27695, USA; dmurato@ncsu.edu; 2 Alltech Inc., Center for Animal Nutrigenomics and Applied Animal Nutrition, 3031 Catnip Hill Road, Nicholasville, KY 40356, USA; ayiannikouris@alltech.com

**Keywords:** aflatoxin, deoxynivalenol, pig, yeast

## Abstract

Pigs are highly susceptible to mycotoxins. This study investigated the effects of a postbiotic yeast cell wall-based blend (PYCW; Nicholasville, KY, USA) on growth and health of newly-weaned pigs under dietary challenge of multiple mycotoxins. Forty-eight newly-weaned pigs (21 d old) were individually allotted to four dietary treatments, based on a three phase-feeding, in a randomized complete block design (sex; initial BW) with two factors for 36 d. Two factors were dietary mycotoxins (deoxynivalenol: 2000 μg/kg supplemented in three phases; and aflatoxin: 200 μg/kg supplemented only in phase 3) and PYCW (0.2%). Growth performance (weekly), blood serum (d 34), and jejunal mucosa immune and oxidative stress markers (d 36) data were analyzed using MIXED procedure of SAS. Mycotoxins reduced (*p* < 0.05) average daily feed intake (ADFI) and average daily gain (ADG) during the entire period whereas PYCW did not affect growth performance. Mycotoxins reduced (*p* < 0.05) serum protein, albumin, creatinine, and alanine aminotransferase whereas PYCW decreased (*p* < 0.05) serum creatine phosphokinase. Neither mycotoxins nor PYCW affected pro-inflammatory cytokines and oxidative damage markers in the jejunal mucosa. No interaction was observed indicating that PYCW improved hepatic enzymes regardless of mycotoxin challenge. In conclusion, deoxynivalenol (2000 μg/kg, for 7 to 25 kg body weight) and aflatoxin B1 (200 μg/kg, for 16 to 25 kg body weight) impaired growth performance and nutrient digestibility of newly-weaned pigs, whereas PYCW could partially improve health of pigs regardless of mycotoxin challenge.

## 1. Introduction

Mycotoxins are fungal secondary metabolites, which may have deleterious effects when consumed. Mycotoxins are naturally present in several plants and products that are used as feedstuffs worldwide, where aflatoxin B1 (AFB1) and deoxynivalenol (DON) are considered major contaminants [1,2]. In the past decade, *Fusarium* toxins were the most prevalent mycotoxins worldwide, where DON ranked first position with 64% of feedstuff samples testing positive, whereas AFB1 was the most frequent among non-*Fusarium* toxins with 23% of samples testing positive [3].

As extensively reviewed [4,5,6], the pig is the most sensitive species to multiple mycotoxins, more specifically, aflatoxins, deoxynivalenol, zearalenone, and fumonisins especially in the early stages of production. Among these, aflatoxins and DON combined decrease pig growth, lead to liver damage, reduce enterocyte viability, and compromise immune function [7,8,9]. Aflatoxin B1 and DON present greater cytotoxicity and have caused synergistic damaging effects in porcine kidney cells [10]. Due to the high prevalence and detrimental effects of AFB1 and DON, regulatory limit levels have been established worldwide. For instance, aflatoxin is limited at 20 [11] or 10 μg/kg of feed [12] for growing pigs, whereas DON has advisory levels of 1000 [13] or 900 μg/kg of feed [14] established by the Food and Drug Administration or European Commission, respectively. Thus, there are several strategies attempting to mitigate negative effects of mycotoxins in animals, such as the implementation of prebiotics, probiotics, and adsorbents [15,16].

Components from yeast organisms, subdivided into parietal (cell wall) and intracellular content, can be used as feed additives. The cell wall consists of a carbohydrate network made of glucans, mannans and chitin [17,18], the former having been identified as key components in mycotoxin deactivation [19,20,21] and prebiotic properties [22,23]. The postbiotic yeast cell wall, and more specifically β-d-glucans composing the inner layer portion of this network, have strong adsorbing affinity against AFB1 or zearalenone, but have limited effectiveness toward deoxynivalenol [21,24]. In addition, certain yeast cell wall components, when specifically isolated and purified, can be advantageous as a prebiotic by favoring proliferation of beneficial microorganisms, enhancing gut barrier function, and supporting intestinal health and immune function [25,26]. 

Therefore, it was hypothesized that use of a postbiotic yeast cell wall-based blend in diets with AFB1 and DON would enhance growth, reduce liver damage, and improve immune function of newly-weaned pigs. 

This study aimed at investigating the effects of a postbiotic yeast cell wall-based blend (PYCW; Alltech Inc., Nicholasville, KY, USA) on growth and health of newly-weaned pigs under chronic dietary challenges of AFB1 (43.6 μg/kg) and DON (2163 μg/kg).

## 2. Results

In the results, diets low in mycotoxins (LM) represent those formulated only with conventional feedstuffs whereas diets high in mycotoxins (HM) represent those formulated with feedstuffs including corn dried distillers grains with solubles (DDGS) high in DON and corn high in AFB1. Experimental diets were fed to newly-weaned pigs moved to the research farm in order to start the current study right after weaning.

The mycotoxin analysis performed comparing LM vs. HM diets fed to pigs show that for phases 1 and 2 the average level of AFB1 was 1.0 μg/kg and DON (including acetylated and glycosylated forms) was 676 vs. 2258 μg/kg, respectively. Mycotoxin analyses performed comparing LM vs. HM diets fed to pigs for phase 3 found the level of AFB1 was 1.0 vs. 44.6 μg/kg and DON (including acetylated and glycosylated forms) was 1311 vs. 4635 μg/kg, respectively. The average mycotoxin concentration in the whole experimental period for LM vs. HM was 887 vs. 3050 μg/kg of DON.

There were no differences in initial body weight of pigs among experimental groups. Feeding HM reduced (*p* < 0.05) average daily feed intake (ADFI) for every week and for the overall period, which resulted in reduced (*p* < 0.05) average daily gain (ADG) and body weight for all periods (Table 1). The HM reduced (*p* < 0.05) ADG of pigs during the first week. There was an interaction (*p* < 0.05) for gain to feed (G/F) ratio during the first week of the study, indicating that when PYCW is added to diets, feeding HM increased (*p* < 0.05) G/F. The interaction also indicates that in pigs fed MT, the addition of PYCW tended to increase (*p* = 0.062) G/F during the first week. Feeding HM also tended to increase (*p* = 0.057) G/F during the second week. Feeding diets with PYCW tended to reduce (*p* = 0.056) G/F during the overall experimental period.

Feeding HM reduced (*p* < 0.05) blood serum protein, albumin, and creatinine concentrations on day 35 (Table 2). Feeding diets with PYCW tended to decrease aspartate aminotransferase (AST; *p* = 0.051) and creatine phosphokinase (CPK; *p* = 0.052) concentrations, and blood urea nitrogen (BUN)/creatinine proportion (*p* = 0.088) in blood serum. Feeding diets with PYCW reduced (*p* < 0.05) the proportion of AST/ALT (alanine aminotransferase) and increased (*p* < 0.05) glucose concentration in blood serum of pigs. There was an interaction (*p* < 0.05) for phosphorus concentration in blood serum of pigs, indicating that when there is no PYCW, HM reduced (*p* < 0.05) phosphorus concentration. The interaction also indicates that in MT, the addition of PYCW tended to increase (*p* = 0.063) phosphorus in serum. 

There were no differences for protein carbonyl, malondialdehyde, total glutathione, tumor necrosis factor-alpha (TNF-α), immunoglobulin A (IgA), immunoglobulin G (IgG), or interleukin 8 (IL-8) in jejunal mucosa among pigs from experimental groups (Table 3).

There were significant interactions for apparent ileal digestibility of dry matter (*p* < 0.05), gross energy (*p* < 0.05), and nitrogen (*p* < 0.05) among pigs from experimental groups (Table 4). These interactions indicate that when there is no PYCW, HM reduced (*p* < 0.05) the apparent ileal digestibility of dry matter, gross energy, and nitrogen. The interactions also indicate that within pigs fed LM, the addition of PYCW decreased (*p* < 0.05) the apparent ileal digestibility of dry matter, gross energy, and nitrogen. Feeding HM also decreased (*p* < 0.05) the apparent ileal digestibility of ether extract. 

In addition, feeding HM decreased (*p* < 0.05) crypt depth from mid jejunum, but no other differences were observed for intestinal histomorphology on villus width, villus height to crypt depth ratio, nor on proportion of Ki-67-positive cells (Table 5).

## 3. Discussion

Considering that naturally contaminated feedstuffs were used for diet formulation, a myriad of mycotoxins were detected, suggesting contamination of multiple fungi. Mycotoxin-contaminated corn was added only to the phase 3 diet to model a real scenario in a commercial farm, where pigs may be fed diets with higher mycotoxin levels as they get older. Such a scenario may happen as older pigs are less susceptible to mycotoxins [27] and, thus, feedstuffs potentially contaminated with mycotoxins may be fed to older pigs. The overall concentration of mycotoxins was three-fold higher in HM than in LM diets, aiming to exceed the guidance levels of mycotoxins’ (AFB1 and DON) concentration in the United States (20 and 1000 μg/kg, [11,13]) and Europe (10 and 900 μg/kg, [12,14]) and cause detrimental effects by feeding HM diet to pigs. The advisory levels of DON for swine are 5000 μg/kg in grain, with grain feedstuffs not to exceed 20% of the final diet [28]. This would result in a concentration of 1000 μg/kg of the final diet. As intended in the current study, aflatoxins and DON concentrations in HM surpassed the values preconized for feeding nursery pigs in the United States. In phases 1 and 2, both LM and HM diets were below (1.0 μg/kg) the threshold for AFB1, whereas in phase 3, HM diet was above the threshold (44.6 μg/kg). For DON in phases 1 and 2, HM diet was above (2258 μg/kg) the threshold for the mycotoxin. During phase 3, both LM and HM diets were above (1311 vs. 4635 μg/kg) the threshold for DON. A lower percentage of DON contaminated DDGS was used on phase 3 (20.8%) in comparison to phases 1 and 2 (22%). However, the supplemental mycotoxin (difference between LM and HM) was achieved as intended (supplemental 2000 µg of DON per kg of feed) in the current study (supplemental 2163 µg of DON per kg of feed).

The levels of DON in the current study are considered as the sum of DON and its acetylated and glycosylated forms. Even though the DON-3-glucoside does not seem to have detrimental effects [29] this choice was based on the previously proven ability of the gut microbiome to convert DON-3-glucoside into the toxic form (DON) both in vitro [30] and in vivo [31] in pigs. Regarding the acetylated forms, they may show either less, similar, or more adverse effects than DON [29,32]. Comparable to DON-3-glucoside, 3-acetyl-DON and 15-acetyl-DON can be converted to DON and, thus, were considered together to have similar effects as DON [33]. Nivalenol is also a type B trichothecene, but its conversion to DON has not been documented. Besides, nivalenol presents stronger harmful effects than DON [32,34] and, thus, is considered as a distinct mycotoxin in the current as well as in other scientific publications [35]. 

Zearalenone average concentration in LM and HM diets (44.3 and 96.0 μg/kg of feed) did not surpass the guidance level of 100 μg/kg preconized in Europe [14] for young pigs. Indeed, previous studies show that zearalenone in this range would not affect health and growth of pigs at 7 to 30 kg body weight [35,36,37,38]. There are no regulations concerning zearalenone concentration in swine feed in the United States. The sum of fumonisins B1, B2, and B3 observed in the current study did not surpass the recommended maximum level set by the Food and Drug Administration [13] of 10,000 μg/kg for pigs. Similarly, the sum of fumonisins B1 and B2 detected in diets was below the guidance level of 5000 μg/kg recommended for pigs in Europe [14]. Considering the low level of contamination of zearalenone and fumonisins, their impacts on health and growth of pigs in this study would be insignificant and, therefore, are not discussed further. 

In the present study, it was observed that feeding HM (aflatoxins at 1.0 μg/kg of feed and DON at 2094 μg/kg of feed) caused reduced feed intake in newly-weaned pigs during phase 1. For phases 2 and 3, AFB1 (1.0 and 44.6 μg/kg) and DON (2421 and 4635 μg/kg) levels were higher and reduced feed intake. In high and acute doses, deoxynivalenol is known to cause vomiting and impair feed intake [36]. In low and chronic doses, deoxynivalenol depresses feed intake, especially in pigs, due to reduced peristalsis mediated by local serotonin [37,38] and satiety signaling mediated by peptide YY (peptide tyrosine tyrosine) [39] along with the release of pro-inflammatory cytokines [40,41]. Aflatoxins also modulate cytokine expression by reducing IL-1β and increasing IL-10 [42] and TNF-α when in combination with DON [7]. In a study performed with mice, DON depressed feed intake as early as 2 h after the ingestion in a dose-response manner [39]. The mechanism underlying feed refusal is related to the increase in peptide YY and serotonin plasma levels, leading to satiety perception, as shown in a previous study [43]. The lower energy and nutrient intake, negatively impacted by HM after phase 1, led to impaired animal growth during the entire period, as observed in ADG. Our study is in accordance with results obtained by Chaytor et al. [7], where AFB1 and DON could impair animal growth at 60 and 300 μg/kg of feed, respectively. Current results are supported by our outcomes observed for gross energy and nitrogen apparent ileal digestibility, where feeding HM reduced the digestibility when no PYCW was added. It was previously shown that DON at 10,000 μg/kg of feed is able to reduce digestibility of essential amino acids in pigs [44]. Similarly, the current study illustrates that, by the end of phase three, pigs fed HM (DON at 4635 μg/kg and AFB1 at 44.6 μg/kg of feed) showed impaired energy and nitrogen apparent ileal digestibility in comparison to pigs fed LM (DON at 1311 μg/kg of feed).

Comparing growth performance for phases 1, 2 and 3, feeding HM reduced ADG by 22, 15, and 14%, and ADFI was reduced by 22, 15 and 12%, respectively. This result suggests that HM effects were stronger during phase 1, while animals were recovering from weaning stress [45]. The interaction in ADG during phase 1 shows that feeding diets with mycotoxins decreased pig ADG when the PYCW was not added to diets. Of interest, when PYCW was added, feeding HM did not reduce ADG during phase 1. At the same time, the interaction in G/F shows that pigs fed HM had greater G/F when PYCW was included. Indeed, PYCW tended to improve G/F among pigs fed MT, indicating that PYCW provided improved growth performance in challenged pigs. During the first week of phase 2, animals fed HM showed greater G/F probably due to its lower ADFI (on average 116 g lower). This is a result of an evolutional adaptation, where pigs eating reduced amounts of energy and nutrient have a compensatory improvement in feed efficiency [46]. In the overall period, adding PYCW tended to reduce G/F, and such decrease in efficiency was supported by the reduction of gross energy and nitrogen apparent ileal digestibility when adding PYCW to LM.

It was not possible to distinguish if the inclusion of AFB1-contaminated corn in phase 3 diet solely influenced the variables tested or if AFB1 inclusion showed additive or synergistic activity that could eventually potentialize one or other deleterious effects of mycotoxins. Comparing growth performance for phase 2 and phase 3, the difference between animals fed LM and animals fed HM was similar for ADG (15 and 14%), but the difference widens for ADFI (15 and 12%), and G/F (−1% and 3%). The observed outcomes are likely to be related to characteristics of each group in the beginning of phase 3, instead of AFB1 addition. The lower body weight of pigs from the mycotoxin group, respectively 9 and 11% lower for phase 2 and phase 3, may have influenced such results, as animals with a smaller body size need less energy and nutrients for maintenance and, thus, can direct these for tissue deposition [47].

Besides the aforementioned effects on pig growth, DON can debilitate liver and kidney function [48,49]. In addition, AFB1 has also shown deleterious effects upon liver and mineral balance in pigs [50]. Weaver et al. [8] has revealed that the combination of AFB1 and DON (AFB1 at 150 and DON at 1100 μg/kg of feed) caused liver damage. In the current study, feeding HM reduced albumin and total protein concentration in serum, which may indicate that liver protein synthesis was compromised due to mycotoxin toxicity [51]. Aflatoxins are known to impair cell protein synthesis through inhibition of RNA polymerase activity in the nucleus [52], resulting in reduced cell viability [53]. Taken together, aflatoxin effects could be responsible for the hypoproteinemia observed as well as impaired pig growth. Mycotoxins tended to decrease the ALT, a cytosolic enzyme investigated to assess liver and kidney functions [54]. Even though an increase in ALT would be expected, cases of chronic liver damage are associated with the reduction in serum levels of the enzyme [55], as observed in the current study. The assumption of liver damage is supported by the tendency to decreased concentrations of cholesterol in pigs fed diets with mycotoxins, as cholesterol is mainly synthetized by hepatocytes [56]. An increase in creatinine level in serum is observed in the case of liver failure [57,58], but, unexpectedly, a decrease in serum creatinine in pigs fed diets with mycotoxins was observed in the current study. Creatinine level in serum linearly increases with pig body weight [59], thus, this could be the reason for the higher creatinine level observed in the group fed LM, which was 3.36 kg heavier at d 36 than pigs fed HM. However, considering there were no changes in BUN, BUN/creatinine, or alkaline phosphatase ratio in animals consuming mycotoxins, it is possible to infer that liver function was not greatly affected in the impairment of nitrogen excretion [60,61,62]. The CPK increase in serum can be indicative of severe hepatic [63] or muscular damage [64]. Hence, the tendency for decreased CPK, and reduced AST and AST/ALT promoted by PYCW suggest that the addition of PYCW may have induced a protective effect in the liver and muscle, reducing the release of their enzymes in serum. The relative level between AST and ALT can be a more reliable variable to evaluate chronic liver damage in humans [65]. Such correlation indicating liver damage can also be observed in pigs challenged with bacterial toxin [66] and under mycotoxin challenge [67]. Furthermore, stimulation of protein synthesis and proliferation in muscular cells proportioned by n-butyric acid [68], one of the components in PYCW, could have mitigated the muscular damage caused by mycotoxins.

The reduction in BUN/creatinine along with the increase in glucose suggest that animals fed PYCW could more efficiently utilize protein and carbohydrate sources in feed. Indeed, yeast cell wall supplementation above 0.05% has been shown to play a role in modulating amino acid and glucose levels in blood serum of pigs [69]. Mycotoxins decreased phosphorus levels in serum of animals in the absence of PYCW, but the addition of PYCW mitigated the deleterious effects of mycotoxins on phosphorus levels in pigs fed HM. Mycotoxin damage to kidney and liver [48,70] may have caused the alteration in phosphorus levels, considering that the liver is the main site for cholesterol synthesis, vitamin D precursor, and that both organs are essential for vitamin D activation [71,72]. Reinforcing this line of thought, and as aforementioned, mycotoxins in fact decreased cholesterol level in serum of pigs fed diets with mycotoxins. In addition, previous data have shown toxic effects of aflatoxin on kidney, calcium and vitamin D metabolism in broilers [73]. The PYCW was able to mitigate mycotoxic effects on phosphorus balance, as seen by the tendency towards increased phosphorus levels in serum of pigs fed diets with mycotoxins. However, further investigation into vitamin D levels as well as kidney and liver function would be necessary to determine if the decrease in phosphorus was related to vitamin D metabolism observed in the current study. Of interest, one of the components of PYCW is vitamin C. The vitamin C pool can be depleted under challenging situations as it is involved in the reduction of oxidative stress [74]. Furthermore, vitamin C supplementation in weaned pigs is related to improved immune function [75].

Pigs challenged with DON have shown an increase in inflammatory cytokine expression as IL-8, as well as up-regulation of glutathione peroxidase 2 gene and IgG [76]. In the present study, impacts of mycotoxins were not strong enough to affect oxidative damage markers, pro-inflammatory cytokines, or immunoglobulins in the jejunal mucosa. In a previous study conducted by Pasternak et al. [77], where pigs chronically exposed to DON (3800 μg/kg of feed vs. 4635 μg/kg of feed in the current study), IL-8 in the ileum was the only cytokine upon which effects of mycotoxin presented a trend to increase. Thus, the relative low mycotoxin level used seemed unlikely to promote changes in immunological variables. 

It was possible to observe that ileal apparent digestibility for gross energy and nitrogen were reduced when adding PYCW to LM. Yeast cell wall is mostly composed of β-D-glucans and mannose-oligosaccharides; the latter has been shown to improve nutrient digestibility when supplemented in a concentrated form from 0.1 to 0.2% in diets [78] with the effect being more pronounced during the first two weeks after weaning [79]. The β-glucans have shown to improve nutrient digestibility in weaned pigs when treated with antibiotics [80]. In the current study, however, there was a lack of difference in nutrient digestibility when adding PYCW in pigs fed HM. Alternatively, the current reduction in apparent ileal digestibility of nutrients in feed observed in pigs fed LM with PYCW could be due to reduced digestibility of β-D-glucans derived from yeast non-starch polysaccharides [81].

The decrease in crypt depth was the only noticeable effect in animals consuming mycotoxins. DON might have impaired cell proliferation in the crypts by inhibiting the Wnt/β-catenin pathway [82]. Nivalenol has shown greater impact in jejunal morphology than DON [34] and the ileum is the most affected segment of the small intestine regarding protein synthesis [76,83], which may explain why no major effects on gut morphology were detected. 

Chronic mycotoxin challenge with DON (3050 μg/kg, for 7 to 25 kg body weight) and AFB1 (44.6 μg/kg, for 16 to 25 kg body weight) clearly impaired growth performance, reduced apparent ileal digestibility of nutrients in feeds, and caused mild liver damage in newly-weaned pigs. The postbiotic yeast cell wall-based blend partly reduced liver damage. In pigs not challenged with mycotoxins, the postbiotic yeast cell wall-based blend reduced apparent ileal digestibility of dry matter, gross energy, and nitrogen; whereas in pigs challenged with mycotoxins, the postbiotic yeast cell wall-based blend maintained growth performance and apparent ileal digestibility of all nutrients in feeds.

## 4. Conclusions

Chronic dietary challenge of DON (3050 μg/kg) and AFB1 (44.6 μg/kg) is harmful to newly-weaned pigs, compromising growth and nutrient digestibility. Supplementation with the postbiotic yeast cell wall-based blend could partially overcome the harmful effects of the dietary challenge of multiple mycotoxins on growth and health of weanling pigs.

## 5. Materials and Methods 

The Institutional Animal Care and Use Committee (IACUC) at North Carolina State University (Raleigh, NC, USA) reviewed and approved the protocol of this experiment.

### 5.1. Animals and Experimental Diets

The levels of selected mycotoxins detected in conventional corn DDGS, DON-contaminated DDGS, and aflatoxin-contaminated corn used for diet formulation are presented on Table 6.

Experimental diets were formulated to meet or exceed the nutrient requirements suggested by the National Research Council (NRC) [84] following a three-phase feeding program (Table 7). The use of three dietary phases followed the recommendation of the NRC to meet nutritional requirements of nursery pigs [84]. All experimental diets were sampled (from nine different locations, 2 kg total per diet), with 200 g of each being sent to North Carolina Department of Agriculture (Raleigh, NC, USA) and to the Analytical Services Laboratory of Alltech Inc. (37+™, ISO/IEC 17025:2005 official accreditation (No. 79481) using LC-MS/MS; Alltech Inc., Nicholasville, KY, USA) for analyses of nutrient composition and mycotoxin concentration, respectively (Table 8). The sample processing for quantitative determination of mycotoxin concentration followed the procedures previously described by Jackson et al. [85]. In short, ground and homogenized feed samples had 400 mg subsampled and placed in silanized glass vials for extraction with acetonitrile/water/formic acid (84.0:15.9:0.1, v/v/v) during 18 h. Vials were centrifuged and the supernatant was dried at room temperature for 30 min under nitrogen stream. A mixture of water/acetonitrile/formic acid (95.0:4.9:0.1, v/v/v) with 10 mmol/L of ammonium acetate was used as loading buffer for analysis of 44 mycotoxins by LC-MS/MS.

**Figure 1:**
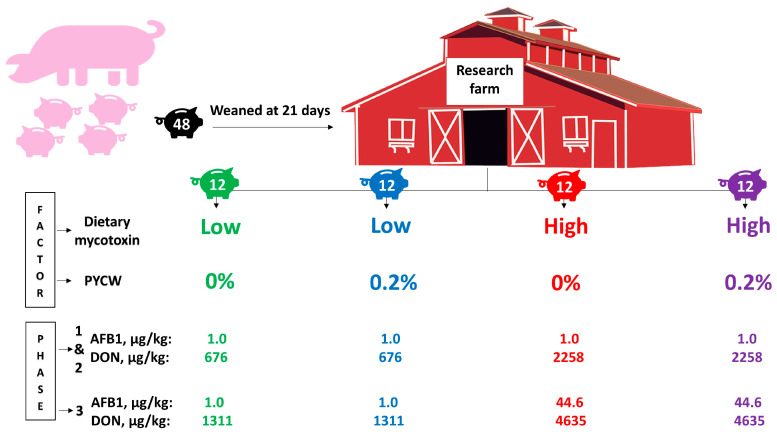
Experimental arrangement and pig assignment to treatments. Forty-eight pigs were weaned at 21 days of age and assigned to four dietary treatments (*n* = 12) following a randomized complete block design in a 2 *×* 2 factorial arrangement. Pigs in green received low mycotoxin diet (LM; formulated without the dietary mycotoxin factor—conventional DDGS and corn); pigs in blue received LM and postbiotic yeast cell wall-based blend (PYCW) at 0.2%; pigs in red received high mycotoxin diet (HM; formulated with dietary mycotoxin factor—DON-contaminated DDGS and, during phase 3, aflatoxin-contaminated corn); and pigs in purple received HM and PYCW at 0.2%. The average aflatoxin B1 (AFB1) and deoxynivalenol (DON) levels during phases 1 and 2, and during phase 3 are specified per treatment.

Forty-eight (24 barrows and 24 gilts) crossbred pigs (PIC 337 × Camborough 22) were weaned at 21 d of age (7.49 ± 0.11 kg). Subsequent to weaning, pigs were moved to the research farm and allotted to four dietary treatments based on a completely randomized block design according to sex and body weight (heavy, medium, and light) with two factors for 36 days based on a three-phase feeding program. Pigs within the heavy body weight group ranged from 9.12 to 7.68 kg, within the medium body weight group the range was 7.64 to 7.28 kg, and within the light body weight group the range was 7.2 to 6.04 kg. The two factors were: (1) dietary mycotoxins, obtained from naturally contaminated DDGS (DON: 887.3 or 3050 μg/kg of feed during all phases) and corn (AFB1: 1.0 or 44.6 μg/kg of feed during phase 3), and (2) PYCW (0 or 0.2%; Alltech Inc., Nicholasville, KY, USA). The PYCW is a proprietary blend of postbiotic functional bioactive constituents containing hydrolyzed yeast cell wall of *Saccharomyces cerevisiae*, organic acids (n-butyric acid), vitamins (ascorbic acid), and essential oils (rosemary extract, Alltech Inc., Nicholasville, KY, USA). An overview of pig assignment to treatments according to the factors and mycotoxin levels per treatment is detailed in Figure 1.

### 5.2. Data Collection

Body weight of pigs and feed consumption by pigs were recorded weekly and used to obtain average daily gain (ADG), average daily feed intake (ADFI), and gain to feed ratio (G/F). On d 35, 10 mL blood samples from the external jugular vein were collected with 0.8 × 32 mm needles (Eclipse, Becton Dickinson Vacutainer Systems, Franklin Lakes, NJ, USA) and serum blood collection tubes (Becton Dickinson Vacutainer Systems, Franklin Lakes, NJ, USA) using a vacutainer tube holder, following procedures routinely employed by our research group [20,86,87]. Serum samples were stored at −80 °C in a freezer (812660-760, Thermo Fisher Scientific, Waltham, MA, USA) in 1.5 mL tubes (Fisherbrand, Fisher Scientific, Hampton, NH, USA) after centrifugation at 1509× *g* at 4 °C for 15 min (5811F, Eppendorf, Hamburg, HH, Germany). Serum samples were used for measuring serum biochemistry (including serum proteins, enzymes, cholesterol, blood urea nitrogen, creatinine, and glucose), and electrolyte profiling (Antech Diagnostics Laboratory, Cary, NC, USA). 

At the end of the study (d 36), pigs were euthanized to obtain scrapped mucosa and intact tissue from mid-jejunum and ileal digesta. Pigs were stunned by captive bolt followed by vena cava exsanguination. Samples of gut mucosa (from 15 cm) from the mid-jejunum were scraped with the aid of clean histological slides. The mid-jejunum of pigs was determined at 3.5 m distal of the duodenum [88]. Mucosal samples were frozen in liquid nitrogen immediately after collection and then transferred to −80 °C until laboratory analyses. Ileal digesta samples were obtained by gently squeezing from the ileocecal junction until the proximal end of the ileocecal fold. Ileal digesta containers were emerged in ice and then stored at −80 °C until laboratory analyses. Tissue samples (5 cm) from the mid-jejunum were placed in 10% buffered formaldehyde at room temperature until further processing for histological evaluation. 

### 5.3. Laboratory Analyses

For protein extraction mid-jejunum mucosa samples were thawed on ice and 1 g of the sample was placed in sterile tube (5 mL tube, Eppendorf, Hamburg, Germany) followed by addition of 2 mL of PBS (MP Biomedicals, Inc., Santa Ana, CA, USA). Samples were homogenized (Tissuemiser, Thermo Fisher Scientific, Waltham, MA, USA) for 30 s and centrifuged at 87,000× *g* for 20 min on ice. The supernatant was subdivided into vials stored at −80 °C until being used to evaluate antioxidant status, immune response, and intestinal barrier function in mid-jejunum mucosa relative to the protein content of samples (PierceTM BCA Protein Assay Kit, Thermo Fisher Scientific, Waltham, MA, USA). Protein quantification started by mixing 25 µL of each sample with 200 µL of working reagent provided in the kit in a microplate well (96-Well EIA/RIA Plates, Corning, Corning, NY, USA), followed by 30 s incubation in plate shaker. The plate was covered with clear adhesive strip and incubated for 30 min at 37 °C. The plate was cooled to room temperature and wells were read at 562 nm. 

The quantification of protein carbonyls (STA-310, Cell Biolabs, Inc., San Diego, CA, USA) as an index of oxidized proteins is described by Shen et al. [89]. Briefly, the protein content of each sample determined in the previous assay was diluted to 10 µg/mL. Diluted samples (100 µL) were pipetted into wells and incubated for 2 h at 37 °C. Each well was washed three times with 250 µL of PBS (MP Biomedicals, Inc., Santa Ana, CA, USA) and 100 µL of working solution supplied in the kit added before plate incubation in the dark for 45 min. Each well was washed with 250 µL of PBS/ethanol (1:1, v/v) and incubated for 5 min in an orbital shaker; this procedure was repeated four times. Each well was washed with 250 µL of PBS twice, 200 µL of blocking solution was added, and the plate was incubated for 1 h in an orbital shaker. Each well was washed with 250 µL of washing buffer three times and 100 µL of anti-dinitrophenylhydrazine antibody supplied in the kit were added according to dilutions recommended by the manufacturer. The plate was incubated in an orbital shaker for 1 h. Each well was washed with 250 µL of washing buffer three times and 100 µL of horseradish peroxidase antibody were added for incubation for 1 h in an orbital shaker. Each well was washed with 250 µL of washing buffer five times, 100 µL of substrate were added, and 100 µL of stop solution were added after the onset color development. The wells were read at 450 nm.

Malondialdehydes (STA-330, Cell Biolabs, Inc., San Diego, CA, USA) were measured by incubating for 5 min 100 µL of each sample in equal volume of SDS lysis solution provided in the kit. Followed by incubation at 95 °C for 45 min with 250 µL of the reagent (130 mg of thiobarbituric acid in 25 mL of diluent) supplied in the kit, which had the pH adjusted (Accumet AB15 pH Meter, Fisher Scientific, Hampton, NH, USA) to 3.5 with sodium hydroxide. Tubes were cooled in for 5 min and centrifuged at 4000× *g* for 15 min. The supernatant (300 µL) was vigorously mixed with 300 µL of butanol for 2 min and centrifuged at 10,000× *g* for 5 min. The supernatant (200 µL) was transferred to a microplate (96-Well EIA/RIA Plates, Corning, Corning, NY, USA) and samples were read at 532 nm. 

Tumor necrosis factor-α (PTA00, R&D Systems, Inc., Minneapolis, MN, USA) was measured by pipetting 50 µL of assay diluent supplied in the kit with 50 µL of samples into wells. The plate was covered with clear adhesive strip and incubated for 2 h. Each well was washed five times with 300 µL of washing buffer, 100 µL of TNF-α conjugate supplied in the kit were added, and the plate was incubated following same specifications. Each well was washed five times with 300 µL of washing buffer, 100 µL of substrate solution supplied in the kit were added to each well, and the plate was incubated for 30 min in the dark. After incubation, 100 µL of stop solution supplied in the kit were added and wells were read 450 and 570 nm to obtain reading at 570 subtracted from 450 nm. 

Iterleukin-8 quantification (P8000, R&D Systems, Inc., Minneapolis, MN, USA) was performed by pipetting 50 µL of assay diluent supplied in the kit with 100 µL of samples into wells. The plate was covered with clear adhesive strip and incubated for 2 h in orbital shaker at 500 rpm. Each well was washed five times with 300 µL of washing buffer, 200 µL of porcine IL-8 conjugate supplied in the kit were added, and the plate was incubated following same specifications. Each well was washed five times with 300 µL of washing buffer, 120 µL of substrate solution supplied in the kit were added, and the plate incubated for 30 min in the dark. After incubation, 120 µL of stop solution supplied in the kit were added and wells were read 450 and 570 nm to obtain reading at 570 subtracted from 450 nm.

Immunoglobulin A (E100-102, Bethyl Laboratories, Inc., Montgomery, TX, USA) and IgG (E100-104, Bethyl Laboratories, Inc., Montgomery, TX, USA) were measured by pipetting 100 µL of their respective affinity purified antibody in each well according to the kit dilution. The plate was incubated for 1 h. Each well was washed five times with 260 µL of washing buffer supplied in the kit, 200 µL of blocking buffer supplied in the kit were added, and the plate was incubated for 30 min. Each well was washed five times with 260 µL of washing buffer, 100 µL of samples were added and incubated for 30 min. Each well was washed five times with 260 µL of washing buffer, 100 µL of diluted horseradish peroxidase supplied in the kit were added, and the plate was incubated for 1 h. Each well was washed five times with 260 µL of washing buffer, 100 µL of tetramethylbenzidine substrate were added, and the plate was incubated in the dark for 15 min. Sulfuric acid (100 µL) at 0.18 M was used as stop solution. The plate was read at 450 nm. 

For measurement of total glutathione, a different protein extraction method was used, as recommended by the kit manufacturer total glutathione (STA-312, Cell Biolabs, Inc., San Diego, CA, USA). Mid-jejunum mucosa (100 mg) and 1 mL of metaphosphoric acid at 5% were mixed and homogenized with a glass pestle. The homogenate was centrifuged at 64,000× *g* for 15 min. The supernatant was used for total glutathione determination total glutathione (STA-312, Cell Biolabs, Inc., San Diego, CA, USA). Glutathione reductase solution (25 µL), NADPH solution (25 µL) supplied in the kit, and samples (100 µL) were added to each well. The chromogen solution (100 µL) supplied in the kit was added to each well and the plate was read at 405 nm every 2 min during 10 min. All wavelengths (for quantifications of protein, protein carbonyls, malondialdehydes, total glutathione, TNF-α, IL-8, IgA, and IgG) were read at the same microplate reader (Synergy HT, Biotek, Winooski, VT, USA).

Ileal digesta was freeze dried (SP Scientific, Virtis 24DX48 GPFD/300820, Warminster, PA, USA) and ground. Subsamples of ground material were analyzed for apparent ileal digestibility of dry matter [90], gross energy (6200 Calorimeter, Parr Instrument Company, Moline, IL, USA), nitrogen (method 990.03, [91], ATC Scientific, North Little Rock, AR, USA), and ether extract (method 920.39, [91]). 

Fixed mid-jejunal tissue was removed from 10% buffered formaldehyde after two weeks for the obtainment of two transversal cuts that were transferred histological cassettes and submerged in 70% ethanol. Mid-jejunal cuts were included in paraffin for assembling histological slides after staining for Ki-67 antigen. The immunohistochemistry staining with Ki-67 primary monoclonal antibody (1:500 dilution) followed by anti-mouse secondary antibody (1:2 dilution factor) and the use of diamino-benzamine reagent for color development was performed in accordance with methods previously described by Kim et. al. [20]. Ten pictures of each pig were used to measure gut morphology by a single researcher choosing a well-oriented villus and its associated crypt. Measurements included: villus width (at half of villus height), villus height (from tip of the villus to top of the crypt), crypt depth (from top to bottom of the crypt), and calculating villus height: crypt depth [86]. The proportion of proliferating cells in the crypt was also estimated by calculating the proportion of cells positive to Ki-67 after taking pictures at 40× in Sony Van–Ox S microscope (Opelco, Washington, DC, USA) and processing in ImageJS tool [92] for analysis as described by Holanda and Kim [86]. 

### 5.4. Data Analyses and Interpretation

The statistical analysis was performed using the mixed procedure of SAS 9.3 software (Cary, NC, USA). Factors, dietary mycotoxins and PYCW, and interaction were considered as main effects, whereas blocks, sex and initial body weight, were considered as random effects. Means were obtained by the LSMEANS statement. In case of interaction, treatments were compared with the PDIFF statement and tested by Tukey test. Results were considered statistically different for *p* < 0.05 and were considered as tendency for 0.05 ≤ *p* < 0.10.

## Figures and Tables

**Table 1:** Growth performance of newly-weaned pigs fed diets high (HM) or low (LM) in mycotoxins ^1^ or postbiotic yeast cell wall-based blend (PYCW) for 36 days.

Diet	LM		HM		SEM	*p* Value		
PYCW	0%	0.2%	0%	0.2%		Diet	PYCW	Diet vs. PYCW
Body weight, kg								
d 0	7.45	7.49	7.51	7.53	0.48	0.659	0.784	0.939
d 7	10.11	9.82	9.33	9.60	0.69	0.016	0.970	0.168
d 14	13.73	13.65	12.47	12.94	0.85	0.005	0.567	0.418
d 21	17.57	17.71	15.76	16.24	0.93	<0.001	0.475	0.692
d 28	22.39	22.31	19.79	20.32	1.29	<0.001	0.697	0.598
d 36	29.49	29.43	25.74	26.47	1.47	<0.001	0.632	0.574
ADG, kg								
Phase 1 (d 0 to 7)	0.379 ^a^	0.333	0.261 ^b^	0.296	0.039	0.001	0.815	0.081
Phase 2 (d 7 to 21)	0.533	0.563	0.459	0.474	0.024	0.001	0.346	0.750
d 7 to 14	0.518	0.547	0.449	0.476	0.031	0.017	0.320	0.980
d 14 to 21	0.549	0.580	0.470	0.472	0.038	0.018	0.667	0.708
Phase 3 (d 21 to 36)	0.794	0.781	0.665	0.682	0.039	<0.001	0.938	0.553
d 21 to 28	0.688	0.657	0.576	0.584	0.055	0.005	0.714	0.545
d 28 to 36	0.887	0.890	0.743	0.768	0.031	<0.001	0.624	0.693
Overall (d 0 to 36)	0.612	0.609	0.506	0.526	0.029	<0.001	0.647	0.547
ADFI, kg								
Phase 1 (d 0 to 7)	0.310	0.315	0.239	0.246	0.040	0.002	0.765	0.972
Phase 2 (d 7 to 21)	0.651	0.700	0.559	0.594	0.039	0.002	0.170	0.817
d 7 to 14	0.622	0.679	0.519	0.551	0.047	<0.001	0.156	0.689
d 14 to 21	0.681	0.721	0.599	0.636	0.040	0.040	0.334	0.972
Phase 3 (d 21 to 36)	1.112	1.129	0.947	1.014	0.067	<0.001	0.253	0.491
d 21 to 28	0.922	0.930	0.790	0.805	0.071	0.003	0.768	0.941
d 28 to 36	1.279	1.302	1.084	1.196	0.069	0.002	0.130	0.319
Overall (d 0 to 36)	0.777	0.804	0.658	0.701	0.048	<0.001	0.191	0.764
G/F								
Phase 1 (d 0 to 7)	1.25	1.06 ^b^	1.11 ^Y^	1.40 ^aX^	0.13	0.381	0.627	0.031
Phase 2 (d 7 to 21)	0.82	0.81	0.83	0.81	0.03	0.963	0.593	0.825
d 7 to 14	0.83	0.82	0.87	0.87	0.04	0.057	0.882	0.726
d 14 to 21	0.81	0.80	0.79	0.75	0.05	0.425	0.557	0.776
Phase 3 (d 21 to 36)	0.72	0.70	0.70	0.68	0.02	0.283	0.164	0.996
d 21 to 28	0.75	0.71	0.73	0.72	0.02	0.842	0.284	0.368
d 28 to 36	0.70	0.69	0.69	0.65	0.03	0.253	0.286	0.616
Overall (d 0 to 36)	0.79	0.76	0.77	0.76	0.02	0.296	0.056	0.507

There was no animal mortality during the experimental period. ^1^ HM has 1.0 μg/kg of aflatoxin B1 (AFB1) and 2258 μg/kg of deoxynivalenol (DON) on average for phases 1 and 2, and 44.6 μg/kg of AFB1 and 4635 μg/kg of DON for phase 3. ^a,b^ Means with different superscripts differ (*p* < 0.05). ^X,Y^ Means with different superscripts tend to differ (0.05 ≤ *p* < 0.10).

**Table 2:** Serum variables observed for serum biochemistry, and electrolytes in newly-weaned pigs fed diets high (HM) or low (LM) in mycotoxins ^1^ or postbiotic yeast cell wall-based blend (PYCW) for 35 days.

Diet	LM		HM		SEM	*p* Value		
PYCW	0%	0.2%	0%	0.2%		Diet	PYCW	Diet vs. PYCW
Total protein, g/dL	5.24	5.26	4.91	4.98	0.15	0.001	0.602	0.742
Albumin, g/dL	3.54	3.51	3.23	3.33	0.13	0.009	0.745	0.489
Globulin, g/dL	1.70	1.75	1.68	1.66	0.14	0.367	0.796	0.605
Albumin/Globulin	2.14	2.05	2.00	2.06	0.22	0.561	0.884	0.513
AST, IU/L	37.3	32.6	43.1	29.3	5.7	0.787	0.051	0.334
ALT, IU/L	28.1	29.3	25.8	25.3	1.7	0.050	0.833	0.562
AST/ALT	1.33	1.14	1.68	1.19	0.22	0.225	0.046	0.370
ALP, IU/L	262	246	249	229	22	0.430	0.346	0.940
CPK, IU/L	5107	2156	4330	2203	1315	0.775	0.052	0.747
Cholesterol, mg/dL	94.3	88.5	84.4	85.4	3.6	0.077	0.508	0.349
BUN, mg/dL	14.3	13.3	14.1	13.2	0.7	0.796	0.126	0.897
Creatinine, mg/dL	1.02	0.99	0.90	0.96	0.04	0.035	0.630	0.232
BUN/Creatinine	14.4	13.6	16.2	13.8	1.1	0.276	0.088	0.413
Glucose, mg/dL	106	111	103	109	2	0.193	0.015	0.829
Phosphorus, mg/dL	11.4 ^a^	11.0	10.3 ^bX^	11.0 ^Y^	0.4	0.019	0.612	0.034
Calcium, mg/dL	11.1	11.0	10.8	11.0	0.2	0.182	0.787	0.334
Sodium, mEq/L	148.7	146.9	149.8	146.0	1.9	0.964	0.145	0.592
Potassium, mEq/L	5.83	5.64	5.81	6.05	0.22	0.370	0.893	0.332
Na/K	26.0	26.4	26.1	24.6	0.9	0.308	0.526	0.264
Chloride, mEq/L	104.8	104.3	106.8	105.0	1.2	0.192	0.252	0.565

^1^ HM has 1.0 μg/kg of AFB1 and 2258 μg/kg of DON on average for phases 1 and 2, and 44.6 μg/kg of AFB1 and 4635 μg/kg of DON for phase 3. AST, aspartate aminotransferase; ALT, alanine aminotransferase; ALP, alkaline phosphatase; BUN, blood urea nitrogen; CPK, creatine phosphokinase. ^a,b^ Means with different superscripts differ (*p* < 0.05). ^X,Y^ Means with different superscripts tend to differ (0.05 ≤ *p* < 0.10).

**Table 3:** Immune and oxidative stress markers from gut mucosa in newly-weaned pigs fed diets high (HM) or low (LM) in mycotoxins ^1^ or postbiotic yeast cell wall-based blend (PYCW) for 36 days.

Diet	LM		HM		SEM	*p* Value		
PYCW	0%	0.2%	0%	0.2%		Diet	PYCW	Diet vs. PYCW
Concentration/mg of protein								
Protein carbonyl, mMol	2.41	2.26	1.97	2.14	0.30	0.206	0.999	0.416
Malondialdehyde, μM	0.382	0.288	0.349	0.350	0.105	0.845	0.536	0.523
Total glutathione, μM	3.02	4.45	4.58	3.74	1.49	0.691	0.784	0.291
TNF-α, pg	4.53	3.86	4.45	5.49	1.19	0.501	0.870	0.458
TNF-α/IgA	3.20	2.32	3.77	2.50	0.93	0.534	0.084	0.753
IgA, μg	2.44	2.15	1.45	2.87	0.74	0.804	0.281	0.108
IgG, μg	1.36	1.48	1.25	1.46	0.31	0.815	0.540	0.885
IL-8, ng	0.255	0.215	0.207	0.270	0.071	0.949	0.825	0.299

^1^ HM has 1.0 μg/kg of AFB1 and 2258 μg/kg of DON on average for phases 1 and 2, and 44.6 μg/kg of AFB1 and 4635 μg/kg of DON for phase 3. TNF-α, tumor necrosis factor-alpha; IL-8, interleukin 8; IgA, immunoglobulin A; IgG, immunoglobulin G.

**Table 4:** Apparent ileal digestibility of dry matter, gross energy, nitrogen, and ether extract in diets high (HM) or low (LM) in mycotoxins ^1^ or postbiotic yeast cell wall-based blend (PYCW) fed to newly-weaned pigs for 36 days.

Diet	LM		HM		SEM	*p* Value		
PYCW	0%	0.2%	0%	0.2%		Diet	PYCW	Diet vs. PYCW
Dry matter, %	66.5 ^ax^	55.9 ^y^	53.4 ^b^	53.7	2.2	0.001	0.023	0.018
Gross energy, %	70.0 ^ax^	59.4 ^y^	56.6 ^b^	56.6	2.0	<0.001	0.011	0.011
Nitrogen, %	80.4 ^ax^	74.9 ^y^	72.6 ^b^	74.1	1.1	<0.001	0.065	0.002
Ether extract, %	98.2	97.6	97.1	96.7	0.4	0.009	0.179	0.888

^1^ HM has 1.0 μg/kg of AFB1 and 2258 μg/kg of DON on average for phases 1 and 2, and 44.6 μg/kg of AFB1 and 4635 μg/kg of DON for phase 3. ^a,b^ Means with different superscripts differ (*p* < 0.05). ^x,y^ Means with different superscripts differ (*p* < 0.05).

**Table 5:** Intestinal morphology and Ki-67 ^1^ proportion in histology sections of mid jejunum in newly-weaned pigs fed diets high (HM) or low (LM) in mycotoxins ^2^ or postbiotic yeast cell wall-based blend (PYCW) for 36 days.

Diet	LM		HM		SEM	*p* Value		
PYCW	0%	0.2%	0%	0.2%		Diet	PYCW	Diet vs. PYCW
Villus width, µm	420.9	432.4	401.9	407.7	7.1	0.263	0.684	0.611
Villus height (V), µm	153.2	157.7	161.2	160.7	19.8	0.100	0.512	0.826
Crypt depth (C), µm	214.0	223.4	203.4	203.5	11.6	0.045	0.522	0.527
V:C	2.00	1.97	2.02	2.04	0.14	0.651	0.932	0.785
Ki-67 proportion, %	25.93	25.61	27.27	27.44	2.40	0.343	0.963	0.882

^1^ Ki-67 is an estimate of the proliferative rate, calculated based on the proportion of cells positive to Ki-67 staining (immunohistochemistry) to the total number of cells in the crypt. ^2^ HM has 1.0 μg/kg of AFB1 and 2258 μg/kg of DON on average for phases 1 and 2, and 44.6 μg/kg of AFB1 and 4635 μg/kg of DON for phase 3.

**Table 6:** Selected mycotoxins detected in conventional dried distillers grains with solubles (DDGS), deoxynivalenol (DON) contaminated DDGS, and aflatoxin (AF) contaminated corn used for diet formulation to newly-weaned pigs for 36 d.

Mycotoxin, μg/kg	Conventional DDGS	DON DDGS	AF Corn
Aflatoxin B1	0.1	0.1	239.6
Aflatoxin B2	0.5	0.5	30.9
Aflatoxin G1	0.1	0.1	14.8
Aflatoxin G2	0.1	0.1	0.1
Deoxynivalenol	2064	5897	4
3-acetyl-deoxynivalenol	21.7	102.9	2.3
15-acetyl-deoxynivalenol	550	2104	2
Deoxynivalenol-3-glucoside	38.7	11.0	11.0
Nivalenol	49.9	49.9	49.9
Fusarenon-X	2.5	2.5	2.5
Fumonisin B1	479	347	29,773
Fumonisin B2	27	37	5478
Fumonisin B3	5	17	6092
Zearalenone	213	1720	3

Mycotoxin concentrations were measured at the Analytical Services Laboratory of Alltech Inc. Laboratory (37+™, Alltech Inc., Nicholasville, KY, USA). The detection limit was used for variables not detected. Levels of mycotoxins reported considered values above the limit of quantitation of each mycotoxin, the relative standard deviation (<20%), and the signal to noise ratio (>10).

**Table 7:** Composition of experimental diets high (HM) or low (LM) in mycotoxins in a three-phase feeding program fed to newly-weaned pig for 36 d ^1^.

Item	Phase 1 (d 0 to 7)		Phase 2 (d 7 to 21)		Phase 3 (d 21 to 36)	
	LM	HM	LM	HM	LM	HM
Ingredient, %						
Ground corn	14.67	14.67	31.07	31.07	45.80	41.05
**Aflatoxin corn ^2^**	**-**	**-**	**-**	**-**	**-**	**4.75**
**Corn DDGS**	**22.00**	**-**	**22.00**	**-**	**20.81**	**-**
**DON corn DDGS ^3^**	**-**	**22.00**	**-**	**22.00**	**-**	**20.81**
Soybean meal	16.00	16.00	19.00	19.00	28.36	28.36
Whey permeate	20.00	20.00	10.00	10.00	-	-
Cookie meal	10.00	10.00	5.00	5.00	-	-
Poultry meal	6.00	6.00	4.00	4.00	-	-
Blood plasma	5.00	5.00	3.00	3.00	-	-
Fish meal	2.00	2.00	-	-	-	-
Poultry fat	2.00	2.00	3.00	3.00	1.90	1.90
Limestone	0.90	0.90	1.05	1.05	1.09	1.09
Dicalcium phosphate	-	-	0.50	0.50	0.76	0.76
Salt	0.22	0.22	0.22	0.22	0.21	0.21
L-lysine HCl	0.53	0.53	0.51	0.51	0.35	0.35
DL-methionine	0.15	0.15	0.12	0.12	0.04	0.04
L-threonine	0.10	0.10	0.10	0.10	0.03	0.03
Mineral mix	0.15	0.15	0.15	0.15	0.14	0.14
Vitamin mix	0.03	0.03	0.03	0.03	0.03	0.03
Zinc oxide	0.25	0.25	0.25	0.25	-	-
Titanium dioxide	-	-	-	-	0.48	0.48
Calculated composition						
DM, %	91.10	91.10	90.48	90.48	89.57	89.57
ME, kcal/kg	3471	3471	3480	3480	3391	3391
SID Lys, %	1.50	1.50	1.35	1.35	1.23	1.23
SID Thr, %	0.88	0.88	0.80	0.80	0.73	0.73
SID Trp, %	0.25	0.25	0.22	0.22	0.23	0.23
SID Met + Cys, %	0.82	0.82	0.74	0.74	0.68	0.68
Ca, %	0.85	0.85	0.80	0.80	0.71	0.71
STTD P, %	0.47	0.47	0.41	0.41	0.33	0.33
DON ^4^, µg/kg	0	2000	0	2000	0	2000
AF, µg/kg	0	0	0	0	0	200

^1^ Postbiotic yeast cell wall-based blend (PYCW) was added to LM and HM at 0.2% in all phases to create two other dietary treatments. ^2^ Aflatoxin corn, aflatoxin-contaminated corn (mycotoxin concentration: 285 μg/kg of aflatoxins and 41.3 mg/kg of feed of fumonisins). ^3^ Deoxynivalenol (DON) corn dried distillers grains with solubles (DDGS), deoxynivalenol-contaminated corn DDGS (mycotoxin concentration: 8115 μg/kg of feed of deoxynivalenol and 401 μg/kg of feed of fumonisins). ^4^ Deoxynivalenol concentration is reported as sum of deoxynivalenol and its metabolites: 3-acetyl-deoxynivalenol, 15-acetyl-deoxynivalenol, and deoxynivalenol-3-glucoside. Ingredients in bold were included or not (no inclusion is represented by a dash “-“) depending on the dietary treatment. DM, dry matter; ME, metabolizable energy; SID, standard ileal digestibility; and STTD, standard total tract digestibility.

**Table 8:** Selected mycotoxins detected in diets high (HM) or low (LM) in mycotoxins in a 3-phase feeding program fed to newly-weaned pig for 36 d.

Mycotoxin, μg/kg	Phase	1		2		3	
	Diet ^1^	LM	HM	LM	HM	LM	HM
Aflatoxin B1		0.1	0.1	0.1	0.1	0.1	43.7
Aflatoxin B2		0.5	0.5	0.5	0.5	0.5	0.5
Aflatoxin G1		0.1	0.1	0.1	0.1	0.1	0.1
Aflatoxin G2		0.1	0.1	0.1	0.1	0.1	0.1
Deoxynivalenol		501	1534	524	1837	1050	3956
3-acetyl-deoxynivalenol		2.3	28.6	2.3	29.7	2.3	25.2
15-acetyl-deoxynivalenol		145	521	154	543	106	435
Deoxynivalenol-3-glucoside		11	11	11	11	152	220
Nivalenol		49.9	49.9	49.9	49.9	49.9	49.9
Fusarenon-X		2.5	2.5	2.5	2.5	2.5	2.5
Fumonisin B1		130	156	219	239	53	1,289
Fumonisin B2		40.9	41.7	39.1	19.0	1.8	67.5
Fumonisin B3		5.0	5.0	5.0	5.0	5.0	41.3
Zearalenone		3	3	128	242	3	43

Mycotoxin concentrations were measured at the Analytical Services Laboratory of Alltech Inc. Laboratory (37+™, Alltech Inc., Nicholasville, KY, USA). The detection limit was used for variables not detected. Levels of mycotoxins reported considered values above the limit of quantitation of each mycotoxin, the relative standard deviation (<20%), and the signal to noise ratio (>10). ^1^ Postbiotic yeast cell wall-based blend (PYCW) was added to LM and HM at 0.2% in all phases to create two other dietary treatments at each phase.
